# Biomedical Applications of Dental and Oral-Derived Stem Cells

**DOI:** 10.1155/2017/2931054

**Published:** 2017-01-03

**Authors:** Boon Chin Heng, Chengfei Zhang, Xuliang Deng, Yin Xiao, Alessandra Pisciotta, Fahad Kidwai, Thimios A. Mitsiadis

**Affiliations:** ^1^The University of Hong Kong, Pokfulam, Hong Kong; ^2^Peking University, Beijing, China; ^3^Queensland University of Technology, Brisbane, QLD, Australia; ^4^University of Modena and Reggio Emilia, Modena, Italy; ^5^University of Minnesota, Minneapolis, MN, USA; ^6^University of Zurich, Zurich, Switzerland

To date, the oral cavity has yielded a diverse menagerie of adult stem cells that includes dental pulp stem cells (DPSCs), dental follicle stem cells (DFSCs), stem cells from apical papilla (SCAP), stem cells from human exfoliated deciduous teeth (SHED), periodontal ligament stem cells (PDLSCs), and gingival mesenchymal stem cells (GMSCs). These stem cells have various useful applications in clinical dentistry that is dental pulp regeneration after root canal treatment, osseous integration of titanium implants, and maxillofacial and periodontal regeneration. Stem cells-based approaches in clinical dentistry are reviewed by D. Hughes and B. Song, as well as S. Miran et al. within this special issue.

Despite their common embryonic origin from the cranial neural crest and their similar fibroblastic morphology within* in vitro* culture, these dental and oral-derived adult stem cell populations exhibit much divergent characteristics (i.e., differences in their plasticity and proliferative potential) and express variable cell surface markers and transcription factors. In this special issue, C.-M. Kang et al. compared the transcriptional profile of human DPSCs and umbilical cord stem cells (UCSCs) with microarray and qRT-PCR techniques and found similar expression levels of the various canonical mesenchymal stem cell markers. However, gene ontology analyses revealed significant differences in the expression of genes related to cell proliferation, angiogenesis, immune responses, growth factor activities, and signal transduction, between DPSCs and UCSCs, which could indicate divergent propensity to differentiate into various lineages.

Dental and oral-derived stem cells have a number of advantages over other more commonly utilized sources of adult stem cells. Compared to the invasive surgical procedure required for obtaining mesenchymal stem cells from bone marrow, these adult stem cells are readily isolated from biological waste produced during routine dental treatment (i.e., extraction of impacted third molars and deciduous teeth). Several studies have reported that dental and oral stem cells such as SHED possess much more extensive multidifferentiation potential and proliferative capacity compared to other adult stem cells. This is extensively discussed in a review on SHED by V. Rosa et al. within this special issue. Even more surprising are reports of the expression of pluripotency markers such as OCT4, MYC, and SOX2 by some dental and oral-derived stem cells, which are not usually expressed in most other adult stem cell types. Expression of these markers might be indicative of the more extensive plasticity and multilineage differentiation potential of dental and oral-derived stem cells when compared to other sources of stem cells. In this special issue, the study of L. Liu et al. reported that these pluripotency markers were strongly expressed in DPSCs at early passage numbers but were gradually downregulated with extended* in vitro* culture. Subsequently, L. Liu et al. demonstrated that transgenic expression of the human xeroderma pigmentosum group C (XPC) gene through a lentiviral vector could mitigate this age-related decline in pluripotency markers expression by DPSCs.

It is possible that the relatively high baseline expression of pluripotency markers by dental and oral-derived stem cells such as DPSCs could render these adult stem cells more amenable to reprogramming into induced pluripotent stem cells (iPSCs). This is critically examined and discussed in a review by J.-H. Lee and S.-J. Seo within this special issue. The study of H. Okawa et al. utilized iPSCs derived from GMSCs to fabricate scaffold-free osteoinductive cellular constructs. Besides the derivation of iPSCs from dental and oral-derived stem cells, it may also be worthwhile to explore the reverse approach, which is the derivation of dental and oral adult stem cell-like lineages from pluripotent stem cells. This may allow us to recapitulate the developmental pathway of the various differentiated somatic lineages of the oral cavity, thus providing us with an extremely useful tool for studying the underlying genetic and developmental basis of various oral and maxillofacial disorders. In this special issue, Q. Zhu et al. reviewed the potential applications of pluripotent stem cell-derived neural crest stem cells, which resemble the various adult stem cell niches of the oral cavity. The study of G. Sriram et al. demonstrated that human embryonic stem cell-derived fibroblasts could be utilized as an alternative to GMSCs for studying the innate immune response to pathogens related to periodontitis (periodontopathogens).

In view of the various promising biomedical applications of dental and oral-derived stem cells, we have organized this special issue as a platform to highlight some of the recent developments and cutting-edge research in this field. It is hoped that the comprehensive reviews and research articles presented in this special issue would stimulate greater interest amongst the scientific community to pursue this field of research, as well as facilitating the translation of research data into clinical therapy.

*Boon Chin Heng* *Boon Chin Heng* *Chengfei Zhang* *Chengfei Zhang* *Xuliang Deng* *Xuliang Deng* *Yin Xiao* *Yin Xiao* *Alessandra Pisciotta* *Alessandra Pisciotta* *Fahad Kidwai* *Fahad Kidwai* *Thimios A. Mitsiadis* *Thimios A. Mitsiadis*

